# Psychotropic drug use among people with dementia – a six-month follow-up study

**DOI:** 10.1186/2050-6511-14-56

**Published:** 2013-11-07

**Authors:** Maria Gustafsson, Stig Karlsson, Yngve Gustafson, Hugo Lövheim

**Affiliations:** 1 Department of Pharmacology and Clinical Neurosciences, Division of Clinical Pharmacology and Department of Community Medicine and Rehabilitation, Geriatric Medicine, Umeå University, Umeå, Sweden; 2 Department of Nursing, Umeå University, Umeå, Sweden; 3 Department of Community Medicine and Rehabilitation, Geriatric Medicine, Umeå University, Umeå, Sweden

**Keywords:** Psychotropic drugs, Dementia, BPSD, Psychotropic prescribing

## Abstract

**Background:**

Psychotropic drugs are widely used among old people with dementia but few studies have described long-term treatment in this group of patients. The purpose of this study was to explore the long-term use of psychotropic drugs in old people with dementia.

**Methods:**

Data on psychotropic drug use, functioning in the activities of daily living (ADL), cognitive function and behavioral and psychological symptoms were collected at baseline and six months later, using the Multi-Dimensional Dementia Assessment Scale (MDDAS). The data were collected in 2005–2006. Detailed data about the prescribing of psychotropic drugs were collected from prescription records. This study was conducted in 40 specialized care units in northern Sweden, with a study population of 278 people with dementia.

**Results:**

At the start of the study, 229 of the participants (82%) were prescribed at least one psychotropic drug; 150 (54%) used antidepressants, 43 (16%) used anxiolytics, 107 (38%) used hypnotics and sedatives, and 111 (40%) used antipsychotics. Among the baseline users of antidepressants, anxiolytics, hypnotics and sedatives and antipsychotics, 67%, 44%, 57% and 57% respectively, still used the same dose of the same psychotropic drug after six months. Associations were found between behavioral and psychological symptoms and different psychotropic drugs.

**Conclusion:**

Psychotropic drug use was high among people with dementia living in specialized care units and in many cases the drugs were used for extended periods. It is very important to monitor the effects and adverse effects of the prescribed drug in this frail group of people.

## Background

The prescribing of drugs for old people is extensive and often inappropriate [1]. A Swedish study of people living in nursing homes shows that over 70% of the residents had one or more potentially inappropriate prescription according to quality indicators published by the Swedish National Board of Health and Welfare [2].

The inappropriate use of drugs has been associated with an increased risk of hospitalization among old people [3] and studies show that up to 30% of hospital admissions are directly connected to drug-related problems [4,5]. Older people are at increased risk of adverse drug reactions, and older people with dementia are especially vulnerable [6].

Of particular concern is the high risk of adverse effects among old people treated with psychotropic drugs. These drugs are widely used among people with dementia [7,8] despite the fact that this group is particularly at risk of the adverse cognitive effects of drugs with anticholinergic properties, such as antipsychotics and certain antihistamines [8,9]. In addition, serious events such as hospital admission or death are frequent following even short-term use of antipsychotics among people with dementia [10]. Many older people with dementia and neuropsychiatric symptoms can be withdrawn from chronic antipsychotic treatment without deterioration, however, some people could benefit from continuing their antipsychotic medication [11]. Benzodiazepines can cause problems with impaired cognition [12], incident mobility and ADL disability among old people [13]. Benzodiazepines and other hypnotics and sedatives might also worsen sleep apnea syndrome [14] and are therefore contraindicated among people with this condition. Sleep apnea syndrome is common among people with dementia, with reported prevalence’s of around 50% and higher [15,16]. Antidepressants also have several side-effects in individuals with dementia, such as falls [17] and hyponatraemia [18]. Taken together, many psychotropic drugs are considered inappropriate, or should be used for a limited period only, or with caution among older people [19].

Behavioral and psychological symptoms are common among people with dementia [20]. Psychotropic drugs are frequently used in nursing homes to treat these symptoms, [21] despite their limited efficacy in this patient group [22,23]. Only a few studies have described long-term psychotropic treatment among people with dementia [21,24]. The aim of one of these studies was to track changes in the prescribing patterns of antidepressants, antipsychotics, anxiolytics and hypnotics over six months among nursing homes residents in Australia [24]. It was found that treatment with psychotropic drugs was, in most cases, not adjusted over time.

We have previously reported data showing that the use of antipsychotic drugs among people with dementia living in specialized care units was high and the treatment in many cases remained unchanged after six months [25]. The study also showed that people who exhibited aggressive behavior or passiveness, or had mild cognitive impairment were at increased risk of being prescribed antipsychotics [25]. The purpose of the present study was to explore the prevalence, associated factors, including behavioral and psychological symptoms, and long-term use of all psychotropic drugs in old people with dementia living in specialized care units. Previously reported analyses of antipsychotic drugs in particular [25] are not included.

## Methods

### Subjects and settings

Data for this study were taken from a research study concerning the use of physical restraint [26]. This was an intervention study conducted in 2005–2006, which included 40 specialized care units for persons with dementia in nine municipalities in northern Sweden. All specialized care units in these areas were inventoried, i.e. 99 units were contacted - and those units with the highest prevalence of physical restraint use (≥ 20%) were selected for inclusion in the study. Our study population comprised 353 people with dementia, and complete data from baseline and a six-month follow up were obtained for 278 persons. Among these 278 people, the mean age was 82 years and 75% were women. All had a dementia diagnosis, and 23% were prescribed an anti-dementia drug. Records were incomplete for 75 out of the 353 people because of incomplete data (16), death (47) or dropout (12). The study was approved by the Regional Ethical Review Board in Umeå (registration number 02–105).

### Procedures

The assessments were made using the Multi-Dimensional Dementia Assessment Scale (MDDAS) [27]. The member of staff who knew each resident best and were most involved in their care performed the assessments based on observations made over the preceding 7 days.

The scale measures, for example, functioning in the activities of daily living (ADL), cognition, and behavior and psychological symptoms. MDDAS also includes a registration of current drug prescription. The MDDAS has good intra- and inter-rater reliability [27]. The ADL function score ranges from 4–24, where a higher score indicates greater ADL independence. This score is based on the patient’s ability to cope with hygiene, dressing, eating and bladder and bowel control. Cognitive impairment was measured using an assessment scale developed by Gottfries and Gottfries [28]. The scale comprises 27 items that measure a person’s cognitive function. Scores of less than 24 are considered to indicate cognitive impairment, correlating with a sensitivity of 90% and a specificity of 91% [28] to the usual cut-off point, 24/30, of the Mini-mental State Examination (MMSE) [29]. The scale is further subdivided into three groups, mild cognitive impairment (16–23), moderate cognitive impairment (8–15) and severe cognitive impairment (0–7). The MDDAS contains 25 behavioral items and 14 psychological symptom items. Each item is rated on a three-point scale indicating whether the symptom was present at least once a day, once a week, or never during the one-week observation period. These variables are dichotomized between at least once a week and less than once a week in the present study.

The prescription records were collected at the start of the study and six months later. The majority used an automated multidose dispensing service where the person’s drugs are dispensed in one-dose-unit bags for each dose occasion.

In this present study, the prescription records collected earlier were searched in order to identify those patients from the study population who were treated with psychotropic drugs. All patients were listed according to age, sex, and treatment with antidepressants (N06A), anxiolytics (N05B), hypnotics and sedatives (N05C), and antipsychotics (N05A). The WHO ATC (Anatomical Therapeutic Chemical Index) classification system was used. Information about dose and type of antidepressant, anxiolytics, hypnotics and sedatives drugs was collected. Pro re nata (PRN) drugs were not included, as information was lacking about the actual use of these drugs.

### Statistics and calculations

PASW Statistics 18 was used for data handling and statistical calculations. A p-value of < 0.05 was considered statistically significant. A multiple logistic regression model was constructed to find factors independently associated with psychotropic drug use. The behavioral and the psychological symptom items of the MDDAS were grouped and weighted (in each group every symptom was multiplied by the calculated factor loading and then added to the next symptom) according to a factor analysis previously described by Lövheim et al. [30]. The factors were then normalized and included in a logistic regression model that also included background variables (age, sex and level of cognitive impairment). As many of the behavioral and psychological symptoms correlated strongly, the behaviors and symptoms were tested in the regression model in a stepwise procedure, where the behavior that had the strongest bivariate correlation was included first, and all other behaviors and symptoms were included subsequently one by one to see if any of them contributed independently. The behavior and symptom factors were: aggressive behavior, wandering behavior, restless behavior, verbally disruptive/attention-seeking behavior, passiveness, hallucinatory symptoms, depressive symptoms, disoriented symptoms and regressive/inappropriate behavior. Ultimately, all significant behaviors and symptoms were included in a final model, one for each drug group.

McNemars test without Yates correction was used to compare the prevalence of symptoms at baseline and follow-up, among people receiving various psychotropic treatments.

## Results

The characteristics of the study population and the prevalence of psychotropic drug use at the start of the study are presented in Table 1. Two hundred and twenty-nine (82%) of the people were prescribed at least one psychotropic drug. One hundred and fifty (54%) used antidepressants, 131 (47%) used anxiolytics, hypnotics and sedatives, and 111 (40%) used antipsychotics. In addition, 74 people in the study population (27%) were prescribed anxiolytics/ hypnotics/sedatives and an antidepressant drug simultaneously. Sixty-two people (22%) were prescribed anxiolytics/hypnotics/sedatives and an antipsychotic drug simultaneously and 61 people (22%) an antidepressant drug and an antipsychotic drug simultaneously. There were 61 people (22%) who used three or more psychotropic drugs concomitantly.

**Table 1:** Characteristics of study population and prevalence of psychotropic drug use at baseline


Cases, n	278
Women, n (%)	209 (75.2)
Mean age ± SD	82.0 ± 8.0
ADL score (4–24) mean ± SD	12.6 ± 5.4
Cognitive score (0–27) mean ± SD	10.7 ± 7.3
Antidepressant (N06A) use, n (%)	150 (54.0)
Anxiolytics, hypnotics and sedatives (N05B&C) use, n (%)	131 (47.1)
Anxiolytics drug (N05B) use, n (%)	43 (15.5)
Hypnotic and sedative drug (N05C) use, n (%)	107 (38.5)
Antipsychotic drug (N05A) use, n (%)	111 (39.9)
Any psychotropic drug use, n (%)	229 (82.4)

SD = Standard deviation, ADL = Activities of daily living.

Furthermore, among antipsychotics (N05A), antidepressants (N06A), anxiolytics (N05B) and hypnotics and sedatives (N05C), 64 people (23%) were prescribed inappropriate drugs, according to the National Board of Health and Welfare (levomepromazine, clozapine, clomipramine, hydroxyzine, diazepam, flunitrazepam and propiomazine) [19]. Eight people used two inappropriate drugs concomitantly.

### Multiple logistic regression analyses

Multiple logistic regression analyses were performed for three psychotropic drug classes: antidepressants (N06A), anxiolytics (N05B) and hypnotics and sedatives (N05C) (Table 2). Younger participants or people with moderate cognitive impairment (compared to severe cognitive impairment) were at increased risk of being prescribed an antidepressant drug. There was no association between antidepressant drug use and depressive symptoms or any other BPSD factor.

**Table 2:** Multiple logistic regression regarding different psychotropic drug use

	***Odds ratio***	***95% confidence interval***	***p-value***
*Antidepressants (N06A)*			
Male sex	0.727	0.381-1.385	0.332
Higher age	0.932	0.897-0.969	0.000
Moderate cognitive impairment^a^	1.971	1.072-3.622	0.029
Mild cognitive impairment^a^	0.933	0.460-1.894	0.848
*Anxiolytics (N05B)*			
Male sex	0.522	0.181-1.505	0.229
Higher age	0.985	0.939-1.032	0.521
Moderate cognitive impairment^a^	0.606	0.242-1.514	0.283
Mild cognitive impairment^a^	1.331	0.509-3.481	0.560
Verbally disruptive/attention-	2.193	1.389-3.462	0.001
seeking behavior			
*Hypnotics and sedatives (N05C)*			
Male sex	1.769	0.917-3.413	0.089
Higher age	0.932	0.896-0.971	0.001
Moderate cognitive impairment^a^	1.401	0.725-2.705	0.316
Mild cognitive impairment^a^	3.627	1.685-7.810	0.001
Disoriented symptoms	1.545	1.169-2.041	0.002

Model Cox and Snell R^2^: 0.078, concordance between observed and predicted value: 65.4% (antidepressants): 0.070/85.5% (anxiolytics): 0.138/69.9% (hypnotics and sedatives). ^a^Cognitive score ranges from 0–27 points and a score of less than 24 is considered to indicate cognitive impairment. The scale is subdivided into three groups, 0–7 (severe cognitive impairment), 8–15 (moderate cognitive impairment) and 16–23 (mild cognitive impairment). Severe cognitive impairment is reference category.

Those who exhibited verbally disruptive/attention-seeking behavior (a factor consisting of the following symptoms: shrieks and shouts continuously, constantly seeks attention of the staff, interrupted night’s sleep, seeks help, disturbed and restless, complains) were at increased risk of being prescribed an anxiolytic drug.

Three variables were associated with hypnotic and sedative drug use: younger age, mild cognitive impairment (compared to severe cognitive impairment) and disoriented symptoms (a factor consisting of the following symptoms: lies in other patients’ beds, take things from other patients’ boxes and closets and undresses in the dayroom).

A multiple regression analysis was also performed including the symptom interrupted night’s sleep and background variables. No association was found between this symptom and the prescribing of hypnotic and sedative drugs (data not shown).

An earlier study explored the associations between antipsychotics (N05A) and behavioral and psychological symptoms in this study population [25].

### Antidepressant drugs

**Figure 1:**
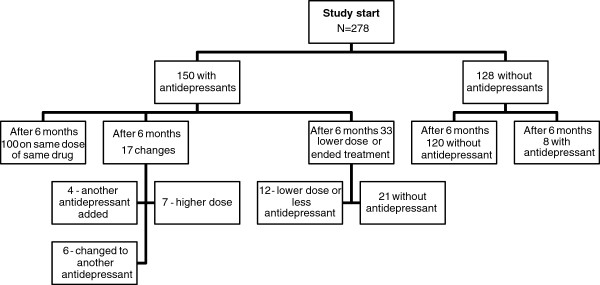
Antidepressants (N06A) – flow chart of participants from baseline to six-month follow-up.

Selective serotonin reuptake inhibitors (SSRI) were the drugs mainly prescribed among the 150 persons who used antidepressant drugs (Table 3). Citalopram accounted for 52% of the antidepressants prescribed followed by sertraline (20%) and mirtazapine (16%). At the start of the study, 150 persons (54%) were prescribed antidepressant drugs (Figure 1). After six months, 100 out of these 150 (67%) were still being treated with the same antidepressant drug in the same dose. Seventeen of these 150 patients showed changes in their prescriptions indicating an escalation of treatment during the six-month period: four people had another antidepressant added, six people had changed to another antidepressant drug and seven had received an increased dose. In total, 33 people had reduced their antidepressant treatment in various ways; 12 had a lower dose or fewer antidepressant drugs, 21 had finished their antidepressant medication completely. This means that 86% (129/150) were still being treated with antidepressant drugs after 6 months. Among the 128 taking no antidepressant drug at the start of the study; eight people were prescribed an antidepressant drug after six months. There was a significant decrease in the proportion of people with at least one of the three depressive symptoms (sad, crying and anxious and fearful) from baseline to the six-month follow-up among people who were treated with antidepressants on both occasions (from 88/120 (73.3%) to 72/120 (60.0%), p = 0.008).

**Table 3:** Characteristics of psychotropic drugs at the start of the study

**Drug**	**n (%)**	**Dose, mean (mg) ± SD**	**Range (mg)**	**Dose, median (mg)**
*Antidepressants (N06A)*				
Citalopram	84 (52.5)	19.6 ± 6.1	10-40	20.0
Clomipramine	1 (0.6)	175.0		175.0
Escitalopram	1 (0.6)	10.0		10.0
Sertraline	32 (20.0)	68.0 ± 31.3	25-150	50.0
Mianserine	8 (5.0)	27.5 ± 10.3	10-40	30.0
Mirtazapine	26 (16.3)	30.6 ± 7.9	15-60	30.0
Venlafaxine	8 (5.0)	112.5 ± 56.7	75-225	75.0
*Anxiolytics (N05B)*				
Hydroxyzine	7 (15.9)	32.9 ± 22.1	10-75	25.0
Diazepam	1 (2.3)	3.3		3.3
Oxazepam	31 (70.5)	24.0 ± 26.8	5-135	15.0
Alprazolam	4 (9.0)	2.6 ± 2.5	0.5-6	2.0
Buspirone	1 (2.3)	15.0		15.0
*Hypnotics and sedatives (N05C)*				
Clometiazol	35 (26.3)	651.4 ± 273.7	300-1500	600.0
Flunitrazepam	15 (11.3)	0.7 ± 0.3	0.5-1	0.5
Propiomazine	37 (27.8)	33.4 ± 12.5	12-50	25.0
Zolpidem	19 (14.3)	6.4 ± 2.5	2.5-10	5.0
Zopiclone	27 (20.3)	6.9 ± 2.2	5-15	7.5

n = number of prescriptions, percent is calculated within each drug group.

### Anxiolytic drugs

**Figure 2:**
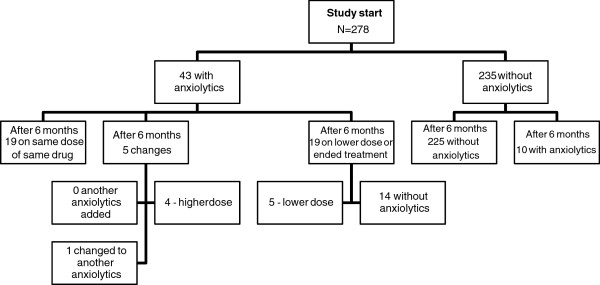
Anxiolytics (N05B) - flow chart of participants from baseline to six-month follow-up.

Concerning anxiolytics, 43 patients (15%) were prescribed such a drug at the start of the study (Figure 2). Oxazepam accounted for 70% of the anxiolytic prescriptions followed by hydroxyzine (16%). After six months, 19/43 patients (44%) were still being treated with the same drug and in the same dose. Five people showed changes in their prescriptions indicating an escalation of treatment during the six-month period, while 19 people had reduced their anxiolytic treatment; 5 were on a lower dose and 14 had ended their anxiolytic treatment, meaning that 67% (29/43) were still being treated with anxiolytic drugs after 6 months. After six months an additional ten people were prescribed an anxiolytic drug. There was no significant change from baseline to follow-up in the proportion of people judged to be anxious and fearful among those treated with anxiolytics on both occasions (data not shown).

### Hypnotic and sedative drugs

**Figure 3:**
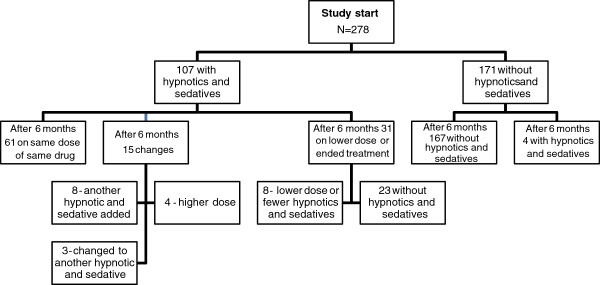
Hypnotics and sedatives (N05C) - flow chart of participants from baseline to six-month follow-up.

A total of 107 persons (38%) in the study population used hypnotic and sedative drugs at the start of the study (Figure 3). Propiomazine (28%) accounted for the largest share of the hypnotic and sedative prescriptions. After six months, 61 of these 107 (57%) were still being treated with the same drug at the same dose. Of these 107 patients, there were changes in the prescriptions of 15 indicating an escalation in treatment during the six-month period. Of these fifteen people, eight had another hypnotic and sedative drug added, three changed to another hypnotic and sedative drug and four were given an increased dose. In total 31 people reduced their treatment; eight had a lower dose or fewer hypnotic and sedative drugs, 23 finished their treatment. In other words, 78% (84/107) were still being treated with hypnotics and sedatives after 6 months. After six months an additional four people were also prescribed a hypnotic and sedative drug. There was no significant change from baseline to follow-up in the proportion of people with interrupted night’s sleep among those treated with hypnotics and sedatives on both occasions (data not shown).

## Discussion

The prevalence of psychotropic drug use (82%) in the study population is in line with or somewhat higher than that reported previously among people with dementia [21,31]. The use of more than one psychotropic drug also seems to be common. This study reveals that many people with dementia who live in specialized care units appear to be on stable doses of psychotropic drugs for six months or possibly longer; after six months, 67%, 44% and 57% were being treated with the same dose of the same antidepressant, anxiolytic or hypnotic/sedative drug respectively. Also, 57% of the study population were being treated with the same dose of the same antipsychotic drug after six months [25].

We found in the present study that more than every second person was prescribed an antidepressant drug (54%) and 67% of those who were prescribed antidepressants were still being treated with the same drug in the same dose after six months. Put another way, for at least 33% changes were made concerning their antidepressant treatment. These results differ from those reported by O’Connor et al. where antidepressants in particular were not adjusted over time. For example in only one out of 59 cases treated in that study was antidepressant medication stopped over the six-month period [24].

We found no association between antidepressant treatment and depressive symptoms. This result also differs from those reported in other studies [7]. The rather small study population might explain this finding, or possibly, these drugs were used also for other indications besides depression – which the high prevalence of antidepressant drugs might indicate. In Sweden, SSRI is recommended as a first-line treatment for irritability, agitation and anxiety among people with dementia [32]. It may also be that people taking an antidepressant drug have no current depressive symptoms because the drug treatment had been successful, therefore few conclusions can be drawn from a lack of association between depressive symptoms and antidepressants. It is much more worrying if persons taking antidepressants are still depressed.

Unlike the situation for all other psychotropic classes, properly monitored, long-term treatment with antidepressants might be appropriate among people with dementia. However, the very high prevalence of antidepressant drug use in this study raises concern. Recent results show that antidepressants have no antidepressant effects among people with dementia, but they do entail an increased risk of adverse events [33]. Nevertheless, we saw a significant decrease in the prevalence of depressive symptoms from baseline to follow-up among people who were treated with antidepressants. However, considering the observational nature of the data, these results have to be interpreted very cautiously. True treatment effects cannot be differentiated from any natural variation in symptoms over time.

Regarding anxiolytics, almost half the patients (44%) were being treated with the same drug and in the same dose after six months. This group includes some of the benzodiazepines (diazepam, oxazepam and alprazolam). These results are in agreement with an earlier study where it was found that benzodiazepines were prescribed with no clear indication for their use and were continued long-term in spite of the risks [34]. Benzodiazepines should not be used for longer than 2–4 weeks by elderly people because of their adverse effects, inducement of dependency and limited efficacy when used continuously [35]. Apart from the long-term use seen in this study, it seems that in most cases a proper benzodiazepine (short-acting) was prescribed.

The high prevalence and long-term use of hypnotic and sedative drugs (38%) in this study warrants concern. More than half the patients (57%) were being treated with the same hypnotic and sedative drug in the same dose after six months. Hypnotic and sedative drugs administered regularly, every night for more than a month, without re-examination are considered as an inappropriate regimen among the elderly according to the Swedish National Board of Health and Welfare [19]. The usefulness and safety of the long-term treatment of elderly people with sleeping pills is not documented. A significant tolerance can be developed to the hypnotic/sedative effect, while the negative effect on psychomotor skills and cognition remains. Intermittent treatment is therefore recommended. Flunitrazepam (long-acting benzodiazepine) should be avoided unless there are special circumstances because of the high risk of side effects in the elderly [19]. Also, given the high prevalence of people with dementia who suffer from sleep apnea syndrome [15,16], treatment with benzodiazepines in this population is in many cases contraindicated. Propiomazine and clometiazol were two commonly used drugs in this study. They were previously manufactured and sold by Swedish pharmaceutical companies and there is a strong tradition of using these drugs in treatment in Sweden. The use of propiomazine among older people, especially those with dementia, is considered inappropriate because of the risk of side effects such as extra pyramidal symptoms. Clometiazol may be used for a short time when urgent sedation is needed and if the patient is adequately monitored [19].

No association was found between the symptom interrupted night’s sleep and the prescribing of hypnotic and sedative drugs, nor did we find any change in the prevalence of interrupted night’s sleep from baseline to six-month follow-up among patients who were treated with hypnotics/sedatives, or in the proportion of people who were anxious and fearful among those taking anxiolytics. This might possibly be a consequence of a tolerance of benzodiazepines and similar drugs already developed at baseline.

The regression analysis shows an association between hypnotics/sedatives and the factor disoriented symptoms and also an association between verbally disruptive/attention-seeking behavior and anxiolytic drugs. The earlier regression analysis with antipsychotics showed that people who exhibited aggressive behavior or passiveness, or had mild cognitive impairment were at increased risk of being prescribed antipsychotics [25]. In another recently published study we found that verbally disruptive/attention-seeking behavior was associated with all psychotropic drug classes, including antipsychotics [7]. Disoriented symptoms and passiveness were also associated with the use of antipsychotics in that study [7]. Taking these findings together might indicate that psychotropic drugs are being used for the wrong reasons in some cases. For example, continuous shouting can be very stressful for the staff. A Dutch study investigated associations between the use of psychotropic drugs and staff distress, aspects of the physical environment and the patient’s neuropsychiatric symptoms, and found an association between staff distress at patient’s agitation and the use of antipsychotic and anxiolytic drugs [36]. Another study from Taiwan found a correlation between the use of psychotropic drugs and the severity of the caregiver’s burden [37]. Concerning aggressive behavior, antipsychotics have been shown to have some efficacy [38], however, evidence of the efficacy of psychotropic drugs for treating patients with verbally disruptive/attention-seeking behavior is limited [23]. Psychotropic drugs with their risk of potentially severe side effects should, however, only be used for the benefit of the person taking them, taking into account both positive and negative effects. Staff distress cannot be regarded as a valid reason for initiating such treatment.

In this study we have been able to describe the long-term use of psychotropic drugs among people with dementia at a detailed level. We found that many people were treated with the same psychotropic drug in the same dose after six months, which indicates long-term treatment among this group of people. Given the significant risk of adverse effects among people with dementia, it is important that proper monitoring of drug therapy is implemented to ensure the appropriate and safe use of medication.

This study has some advantages. The pharmaceutical registration was of high quality since the prescription records were searched in detail. We can also assume that compliance was high since the vast majority of those taking the drugs used an automated dose dispensing system and the staff delivered the drugs.

The study also has some limitations that should be considered. The data were collected in 2005–2006. We cannot know if the prescribing has changed since then, but there is no reason to believe that long-term treatment with psychotropic drugs among people with dementia has changed considerably.

The rather small number of people in the study population may have meant that we did not discover some associations between the psychotropics and behavioral and psychological symptoms. Data were registered at the start of the study and six months later, but we know nothing about what happened between those two dates, for example whether dose reductions were tried and reversed because of aggravated symptoms. We do not know the duration of psychotropic treatment at the time of recruitment into the study, and we do not know the background of the participants or if they had other diseases. Some people might have bipolar disorder or other chronic psychiatric illnesses where long-term treatment of some psychotropic drugs might be appropriate when properly monitored.

Also, the selection of specialized care units was not random but based on the prevalence of the use of physical restraint. It could be that people in these homes have severe problems with BPSD and, therefore, receive long-term treatment of psychotropic drugs to a greater extent. However, comparing this population with an unselected material of persons living in specialized care units for people with dementia (a subset of the material presented in Lövheim et al. 2006) [39], also assessed with the MDDAS, there were no differences concerning the prevalence of aggressive behavior and verbally disruptive/attention-seeking behavior (data not shown). We believe that the selection of participants does not affect the main results of the study, but it should be borne in mind when interpreting the results.

## Conclusion

Psychotropic drug use among people with dementia living in specialized care units was high and in many cases the drugs seemed to be used for extended periods. It is very important to monitor the effects and adverse effects of the prescribed drug in this frail group of people.

## Competing interests

The authors declare that they have no competing interests.

## Authors’ contributions

SK and YG were responsible for the study concept, design and acquisition of subjects. MG reviewed the data a second time and MG and HL made the statistical analysis. MG and HL analyzed and interpreted the data and prepared the manuscript. All authors critically revised the manuscript, added their comments and approved the final version.

## Pre-publication history

The pre-publication history for this paper can be accessed here:

http://www.biomedcentral.com/2050-6511/14/56/prepub
